# Intelligent Traffic Flow Prediction and Analysis Based on Internet of Things and Big Data

**DOI:** 10.1155/2022/6420799

**Published:** 2022-06-15

**Authors:** Bing Liu, Tao Zhang, Weicheng Hu

**Affiliations:** ^1^Department of Information Engineering, Tongling Polytechnic, Tongling 244061, Anhui, China; ^2^College of Mathematics and Computer Science, Tongling University, Tongling 244061, Anhui, China

## Abstract

Nowadays, the problem of road traffic safety cannot be ignored. Almost all major cities have problems such as poor traffic environment and low road efficiency. Large-scale and long-term traffic congestion occurs almost every day. Transportation has developed rapidly, and more and more advanced means of transportation have emerged. However, automobile is one of the main means of transportation for people to travel. In the world, there are serious traffic jams in almost all cities. The excessive traffic flow every day leads to the paralysis of the urban transportation system, which brings great inconvenience and impact to people's travel. Various countries have also actively taken corresponding measures, i.e., traffic diversion, number restriction, or expanding the scale of the road network, but these measures can bring little effect. Traditional intelligent traffic flow forecasting has some problems, such as low accuracy and delay. Aiming at this problem, this paper uses the model of the combination of Internet of Things and big data to apply and analyze its social benefits in intelligent traffic flow forecasting and analyzes its three-tier network architecture model, namely, perception layer, network layer, and application layer. Research and analyze the mode of combining cloud computing and edge computing. From the multiperspective linear discriminant analysis algorithm of the combination method of combining the same points and differences between data and data into multiple atomic services, intelligent traffic flow prediction based on the combination of Internet of Things and big data is performed. Through the monitoring and extraction of relevant traffic flow data, data analysis, processing and storage, and visual display, improve the accuracy and effectiveness and make it easier to improve the prediction accuracy of overall traffic flow. The traffic flow prediction of the system of Internet of Things and big data is given through the case experiment. The method proposed in this paper can be applied in intelligent transportation services and can predict the stability of transportation and traffic flow in real time so as to optimize traffic congestion, reduce manual intervention, and achieve the goal of intelligent traffic management.

## 1. Introduction

At present, almost all major cities have problems such as poor traffic environment [1] and low road efficiency. Large area and long-term traffic congestion occurs almost every day. The extremely congested traffic every day leads to the increase of people's travel time and transportation costs and a great waste of time, energy, and money and increases the pressure of urban life [2]. In the development of urbanization [3], traffic congestion [4] is one of the main urbanization problems that need to be solved. The application of Internet of Things technology [5,6] and big data technology [7] in traditional industries has produced certain economic benefits. In the application of smart transportation and the construction of smart cities [8], traffic data can be quickly collected by using Internet of Things technology, and traffic flow prediction can be realized by using big data technology. Sensor technology [9], as an important data acquisition source in the Internet of Things, can realize fast and stable data transmission and fast storage and analysis [10]. Intelligent transportation can rely not only on the algorithm model [11], but also on the historical data of road traffic. Big data technology is used to process large-scale data and high-dimensional data [12] and capture the data in real time, quickly and effectively. Big data has four characteristics: magnanimity, diversity, efficiency, and variability. The combination of these four characteristics provides basic support for traffic flow data. Establish the corresponding intelligent traffic flow data analysis system based on big data analysis, extract the historical data of road traffic from the specific traffic system [13], analyze and process these data to form structured traffic flow data, and then store the analyzed structural data in the corresponding target server database [14]. Finally, real-time, effective, and accurate traffic flow data are obtained through Internet of Things technology. This system can quickly analyze the changes of various types of data, accurately predict these changes, and screen the information accordingly. In the intelligent traffic flow prediction model based on intelligent data analysis and application, intelligent technology integration is realized through Internet of Things technology and sensor application [15], such as fiber Bragg grating sensor [16], positioning technology [17], RFID application [18], and wireless sensor technology [19]. How to use big data technology and Internet of Things technology in the field of intelligent transportation has become an important technical means of transportation application in the future. Through intelligent transportation technology, traffic accidents can be handled immediately, cost can be saved, and the system can respond quickly and be handled in time. Under the Internet of Things technology, wireless network information has a strong antiinterference and high positioning accuracy. Therefore, the application of big data and Internet of Things technology in traffic flow forecasting can alleviate urban traffic pressure and is one of the important indicators in realizing smart cities. Therefore, Internet of Things and big data can be widely used in modern cities, and the development of traditional industries can also be realized.

## 2. Application of Internet of Things and Big Data in Intelligent Traffic Flow Prediction

### 2.1. Traffic Flow Characteristics

The transportation system is huge and complex, and there are many factors that can cause the poor operation state of traffic flow, such as the changeable weather and the occurrence of accidents. Small changes in the external environment can have a great impact on it, and the performance state of each face is complex, random, and irregular, with obvious dynamic characteristics. Therefore, it becomes very difficult to accurately predict. Hence, it is necessary to comprehensively consider the prediction and analysis of traffic flow, generally from spatial and traffic timeliness. The spatial characteristics of traffic flow means that when observing traffic flow, the parameters of traffic flow at the observation site are inseparable and the traffic state at the observation site is generally directly affected by the overall traffic flow. When the distance of the predicted road section increases, the influence degree decreases; when the distance of the predicted road section decreases, the degree of impact becomes greater.

According to the traffic flow in the past, the change has periodicity, which exists every day, every week, every quarter, and even every year. Looking at the traffic flow every day, it is not in a balanced state as a whole, but obviously has great fluctuations, such as morning peak and evening peak. During these two peak periods, the traffic flow and road congestion are extremely serious compared with the normal time, which is unmatched by the traffic flow in general time. Similarly, if one year is used to analyze the periodicity of traffic flow, the periodicity of traffic flow in each quarter is also very different. Therefore, the traffic flow will roughly show periodic changes due to external factors. Therefore, it is precisely because the traffic flow shows periodic characteristics that the prediction of traffic flow becomes feasible.

The randomness in the time characteristics of traffic flow is mainly reflected in the short-term traffic flow. In the whole transportation system, people mainly manipulate vehicles, whether drivers driving or pedestrians walking on the road, which will affect the whole transportation system. People's behavior cannot be predicted. Sometimes just because of a small action to run the red light, it may cause an irreparable situation; the diversity of vehicle types will also lead to the change of traffic flow. These are unpredictable events, which makes the randomness of traffic flow stronger. Randomness is also a factor that must be considered in real traffic prediction, which increases the difficulty of traffic flow prediction.

**Figure 1:**
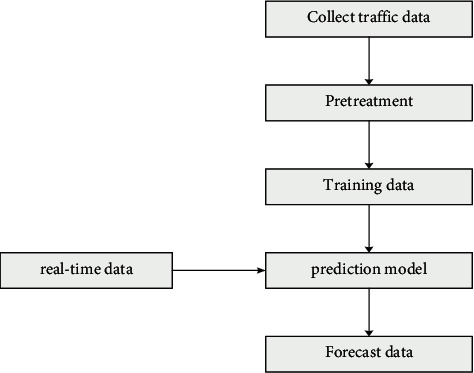
Traffic flow prediction process.

Jiao tong traffic flow prediction will predict the traffic flow data of the road section in the past period, process and analyze the real-time dynamic data collected by wireless sensors and other devices, and use the characteristics of the data to predict the traffic flow of the current road section in the future, as shown in Figure 1.

To predict the circulation flow in the future, it must be based on historical data. Firstly, historical traffic data are collected for data preprocessing, the system starts training the data, calculates the prediction model through real-time data, obtains the prediction data, and finally converts the analysis results into charts and other visual results to respond to users.

#### 2.1.1. Traffic Flow Prediction Method

**Figure 2:**
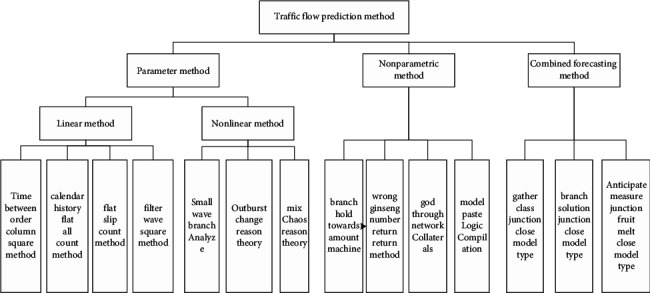
Classification of traffic flow forecasting methods.

Now, there are generally three methods to realize traffic flow prediction, namely, parameter prediction method, nonparametric prediction method, and combined parameter prediction method, which are shown in Figure 2.

Traffic flow forecasting methods are divided into parametric methods, nonparametric methods, and combined forecasting methods. Among them, parametric methods can be divided into linear and nonlinear styles. The linear method can be subdivided into time series method, historical average algorithm, smoothing algorithm, and filtering algorithm; nonlinear methods can be divided into wavelet analysis, catastrophe theory, and chaos theory; nonparametric methods include support vector machine, nonparametric regression, neural network, and fuzzy logic; combined forecasting method is the combination of two or more forecasting methods to achieve the effect of joint forecasting. It adopts the clustering combination model, decomposition combination model, and prediction result fusion model technology.

### 2.2. Intelligent Transportation Technology in Internet of Things and Big Data

**Figure 3:**
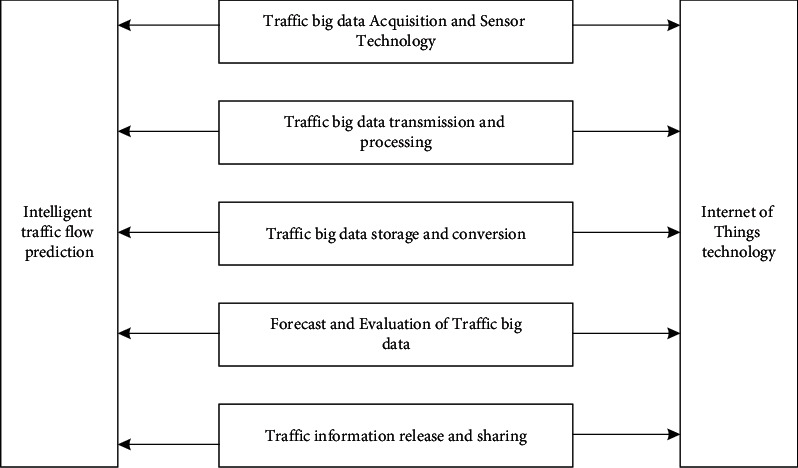
Intelligent transportation technology in Internet of Things and big data.

The application of Internet of Things technology in intelligent transportation has become the main way to relieve traffic pressure. Integrating Internet of Things technology into intelligent traffic flow prediction can make traffic roads smarter and make cities develop faster and more harmoniously. The key technologies are shown in Figure 3.

In Figure 3, intelligent transportation technology in Internet of Things mainly includes data collection, data analysis, data processing, data storage, and data sharing.

The problem to be solved in the road traffic flow data analysis service environment is how to timely analyze the diverse characteristics of traffic flow data, so as to provide accurate services. It should have the ability to enhance service value, mine data value, and reduce response time.

**Figure 4:**
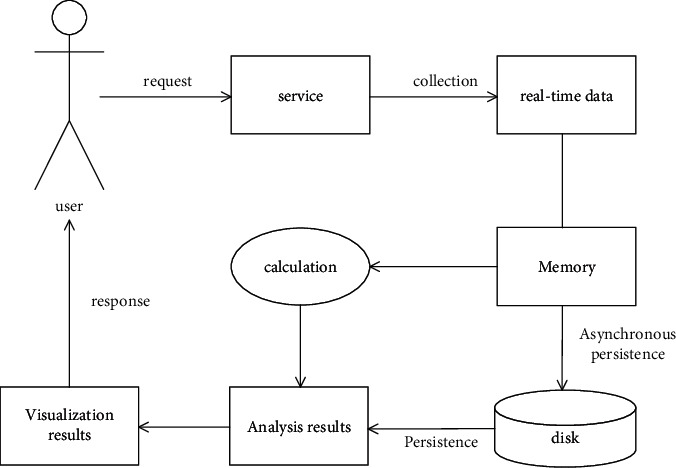
Online big data service mode.

**Figure 5:**
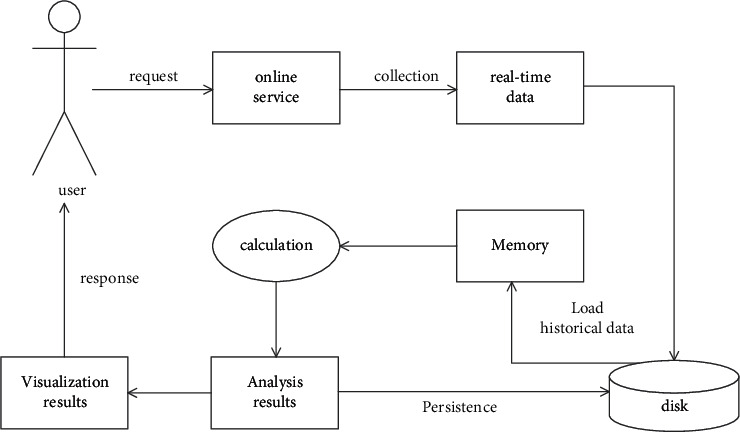
Offline big data service mode.

After big data analysis, it will analyze, process, and calculate the object data according to relevant algorithms and provide the service. The basic process of the big data analysis service mode is shown in Figure 4 (online mode) and Figure 5 (offline mode).

This mode has high real-time requirements and slightly small data throughput. The collected data will not be stored, and the results will be calculated directly in memory. Finally, the analysis results will be directly transformed into visual results to respond to the user. For the offline big data service mode, the biggest difference from the online mode is that it will store the collected historical data and real-time data in the disk. When the data needs to be processed, it will load, process, and calculate in the memory, and finally convert the analysis results to the user. This offline service mode is characterized by low real-time requirements and deep mining of data.

### 2.3. Multiperspective-Based Learning Method

For the same thing, if multiple people describe it, then each person's description methods will be different. Hence, describing the same data from different angles or ways is the method of multiperspective data learning. Data have multiple feature sets. The data obtained from multiple feature sets have different attributes, and these attributes are revealed from different levels and different perspectives. Such data are called multiperspective data. In multiview data learning, there are various analysis technologies, among which the canonical correlation analysis (CCA) and collaborative training technology are the most representative.

CCA is a multivariate statistical method of the cross-covariance matrix, which reflects the overall correlation through the correlation between various variables. *U*_1_ and *V*_1_ represent two sets of variables and are linearly combined to reveal the dependence of the variables on the whole. According to relevant research, canonical correlation analysis acts on the data represented by two or more perspectives and finds two linear transformations for each perspective. Assuming that there are data sets {(*x*_1_, *y*_1_), *···*, (*x*_*m*_, *y*_*m*_)} and  *X*=[*x*_1_, *···x*_*m*_], *Y*=[*y*_1_, *···y*_*m*_], the formula is as follows:

(1) $$\begin{array}{c} \frac{\text{cov}\left(w_{x}^{T}Xw_{y}^{T}Y\right)}{\sqrt{{\text{varw}}_{x}^{T}\text{var}W_{y}^{T}Y}}=\frac{w_{x}^{T}C_{xy}w_{y}}{\sqrt{\left(w_{x}^{T}C_{xx}w_{x}\right)\left(w_{y}^{T}C_{yy}w_{y}\right)}}, \end{array}$$

*w*_*x*_ and *w*_*y*_ are the two projection directions to maximize the linear correlation coefficient, and *C*_*xy*_ is defined as follows:

(2) $$\begin{array}{c} C_{xy}=\frac{1}{m}\overset{m}{\underset{t=1}{\sum }}\left(x_{1}-m_{x}\right){\left(y_{1}-m_{y}\right)}^{T}, \end{array}$$

*m*_*x*_ and *m*_*y*_ are the average of the two perspectives, defined as follows:

(3) $$\begin{array}{c} m_{x}=\frac{1}{m}\overset{m}{\underset{t=1}{\sum }}x_{1},\\ m_{y}=\frac{1}{m}\overset{m}{\underset{t=1}{\sum }}y_{1}. \end{array}$$

That is, the objective function of typical correlation analysis technology can be obtained:

(4) $$\begin{array}{c} \text{max}\left(w_{x},w_{y}\right)w_{x}^{T}C_{xy}w_{y},\\ s.t.\;w_{x}^{T}C_{yy}w_{y}=1. \end{array}$$

The corresponding Lagrange function is as follows:

(5) $$\begin{array}{c} L\left(w_{x^{y}}w_{y^{y}}\lambda_{x}\lambda_{y}\right)=w_{x}^{T}C_{xy}w_{y}-\frac{\lambda_{x}}{2}\left(\;w_{x}^{T}C_{xx}w_{x}-1\right)-\frac{\lambda_{y}}{2}\left(\;w_{y}^{T}C_{yy}w_{y}-1\right). \end{array}$$

If for *w*_*x*_ and *w*_*y*_, take the derivative respectively and make it 0, then the formulas are as follows:

(6) $$\begin{array}{c} C_{xy}w_{y}-\lambda_{x}C_{xx}w_{x}=0,\\ C_{yx}w_{x}-\lambda_{y}C_{yy}w_{y}=0. \end{array}$$

Using these two formulas, we can get

(7) $$\begin{array}{c} \lambda_{y}\;w_{y}^{T}C_{yy}w_{y}-\lambda_{x}w_{x}^{T}C_{xx}w_{x}=\lambda_{y}-\lambda_{x}=0. \end{array}$$

*λ*_*y*_=*λ*_*x*_, If *λ*_*y*_ − *λ*_*x*_=*λ*, *C*_*yy*_ is reversible. Then you can get *w*_*y*_:

(8) $$\begin{array}{c} w_{y}=\frac{1}{\lambda }C_{yy}^{-1}C_{yx}w_{x} \end{array}$$

Similarly, the generalized eigenvalue decomposition problem can be obtained as follows:

(9) $$\begin{array}{c} C_{xy}C_{yy}^{-1}C_{yx}w_{x}=\lambda^{2}C_{xx}w_{x}. \end{array}$$

Finally, in order to make *λ*^2^, if the relationship between the eigenvalue and the correlation coefficient is clearer, the rewriting objective function is defined as follows:

(10) $$\begin{array}{c} w_{x}^{T}C_{xy}w_{y}=\frac{1}{\lambda }w_{x}^{T}C_{xy}C_{yy}^{-1}C_{yx}w_{x},\\ \frac{1}{\lambda }w_{x}^{T}\lambda^{2}C_{xx}w_{x}=\lambda w_{x}^{T}C_{xx}w_{x}=\lambda . \end{array}$$

You can get

(11) $$\begin{array}{c} w_{x}^{T}C_{xy}w_{y}=\frac{1}{\lambda }w_{x}^{T}C_{xy}C_{yy}^{-1}C_{yx}w_{x}=\frac{1}{\lambda }w_{x}^{T}\lambda^{2}C_{xx}w_{x}=\lambda w_{x}^{T}C_{xx}w_{x}=\lambda . \end{array}$$

*λ* is a positive correlation solution which affects the correlation of the projection; therefore, the range of *λ* must vary between [−1, +1].

Finally, according to the calculation results, the biggest feature of using the canonical correlation analysis is that there will be an overfitting of relevant features, and the normalized useful mode will be used, i.e., the function is defined as follows:

(12) $$\begin{array}{c} \frac{w_{x}^{T}C_{xy}w_{y}}{\sqrt{\left(\left(1-\tau_{x}\right)w_{x}^{T}C_{xx}w_{y}+\tau_{x}{\left\|w_{x}\right\|}^{2}\right)\left(\left(1-\tau_{y}\right)w_{y}^{T}C_{yy}w_{y}+\tau_{y}\tau_{x}{\left\|w_{x}\right\|}^{2}\right)}}, \end{array}$$

where *τ*_*x*_ and *τ*_*y*_ are in the change of [0, 1], and the latest statistical analysis believes that regularization is a reasonable standard method to control the projection direction, which will maximize the correlation between the two perspectives and minimize the difference in square loss in each perspective.

### 2.4. Prediction of Traffic Flow

The so-called traffic flow prediction, through the use of information technology on historical data, finds the rules and predictions of traffic operation, thus effectively reducing communication, congestion, and accidents.

In this paper, the Markov model is used to effectively study and analyze the historical data of AC traffic.

In the Gaussian–Markov model, vectors show normal characteristics.

Mathematical expectations are as follows:

(13) $$\begin{array}{c} E\left(\varepsilon \right)=0. \end{array}$$

The variance covariance matrix is as follows:

(14) $$\begin{array}{c} E\left(\varepsilon \varepsilon^{\prime }\right)=\sigma^{2}I△\text{Cov}\left(\varepsilon \right),\\ \varepsilon ∼N\left(0,\sigma^{2}I\right). \end{array}$$

By calculating the expected value of the random vector *Y* for formula (1):

(15) $$\begin{array}{c} E\left(Y|X\right)=XB. \end{array}$$

The variance covariance matrix is as follows:

(16) $$\begin{array}{c} \text{Cov}\left(Y\right)=\sigma^{2}I, \end{array}$$

(17) $$\begin{array}{c} E\left(b_{i}\right)=B_{i}. \end{array}$$

In formula (17), *b*_*i*_ is *B*_*i*_ of unbiased estimation.

(18) $$\begin{array}{c} \text{Var}\left(b_{i}\right)=\frac{\sigma^{2}}{1-R_{i}^{2}}. \end{array}$$

In formula (18), *R*_*i*_^2^ is the negative correlation coefficient.

K-order multivariate linear regression is defined as follows:

(19) $$\begin{array}{c} \left\{\begin{array}{c} x_{11},x_{21},\ldots ,x_{k1},y_{1}\\ x_{12},x_{22},\ldots ,x_{k2},y_{2},\\ .\\ .\\ .\\ x_{1i},x_{2i},\ldots ,x_{ki},y_{i},\\ .\\ .\\ .\\ x_{1n},x_{2n},\ldots ,x_{kn},y_{n}. \end{array}\right. \end{array}$$

The regression model is defined as follows:

(20) $$\begin{array}{c} y_{i}^{*}=b_{0}+\overset{k}{\underset{i=1}{\sum }}b_{i}x_{it}. \end{array}$$

The least squares method is defined as follows:

(21) $$\begin{array}{c} \left(\begin{array}{c} S_{11}\;S_{12}\;\cdot \cdot \cdot \;S_{1k}\\ \begin{array}{c} S_{21}\;S_{22}\;\cdot \cdot \cdot \;S_{2k}\\ \vdots \vdots \vdots \vdots \\ S_{k1}\;S_{k2}\;\cdot \cdot \cdot \;S_{1kk} \end{array} \end{array}\right)\left(\begin{array}{c} b_{1}\\ \begin{array}{c} b_{2}\\ \vdots \\ b_{k} \end{array} \end{array}\right)=\left(\begin{array}{c} S_{1k}\\ \begin{array}{c} S_{2y}\\ \vdots \\ S_{ky} \end{array} \end{array}\right). \end{array}$$

## 3. Optimization and Improvement Algorithm

### 3.1. Kalman Filtering Algorithm

The Kalman algorithm is realized by using a recursive method to deduce the previous state and the current state of the data. According to the existing prediction model, the Kalman algorithm shows the state and observation equation:

(22) $$\begin{array}{c} X_{k}=F_{k/k-1}X_{k-1}+G_{k-1}W_{k-1},\\ L_{k}=H_{k}X_{k}+V_{k}. \end{array}$$

*W*_*k*_ is dynamic noise, *V*_*k*_ is the observation noise sequence, and its statistical vector is defined as follows:

(23) $$\begin{array}{c} \left\{\begin{array}{c} E\left[W_{k}\right]=0,\\ E\left[V_{k}\right]=0, \end{array}\right.\\ \left\{\begin{array}{c} E\left[W_{k}W_{j}^{T}\right]=Q_{k}\delta_{kj},\\ E\left[V_{k}V_{j}^{T}\right]=R_{k}\delta_{kj}, \end{array}\right. \end{array}$$

where *Q*_*k*_ is the variance matrix of dynamic noise,*R*_*k*_ is the variance matrix of observation noise, and *δ*_*kj*_ is the Kronecker function.

(24) $$\begin{array}{c} \delta_{kj}=\left\{\begin{array}{c} 0,\left(k\neq j\right),\\ 1,\left(k=j\right), \end{array}\right.\\ E\left[W_{k}V_{j}^{T}\right]=0,\\ \left\{\begin{array}{c} \hat{X_{0}}=E\left(X_{0}\right)=\mu x_{0},\\ \hat{P_{0}}=\text{Var}\left(X_{0}\right). \end{array}\right. \end{array}$$

*X*_0_ is the set initial value, and  the relation between *W*_*k*_ and *V*_*k*_ denotes independence.

(25) $$\begin{array}{c} \left\{\begin{array}{c} E\left[X_{0}W_{j}^{T}\right]=0,\\ E\left[X_{0}V_{j}^{T}\right]=0. \end{array}\right. \end{array}$$

If set target initial state *X*_0_, *P*_0_ is OK, then get *X*_*t*_ Tminimum square difference.

(26) $$\begin{array}{c} {\tilde{Z}}_{k/k-1}=Z_{k}-H\left(k\right){\hat{X}}_{k/k-1},\\ S_{k/k-1}=E\left[{\tilde{Z}}_{k/k-1}{\tilde{Z}}_{k/k-1}^{r}\right]=H\left(k\right)P_{k/k-1}H{\left(k\right)}^{r}+R_{k}. \end{array}$$

Calculated gain is as follows:

(27) $$\begin{array}{c} K_{k/k}=P_{k/k-1}H_{k}^{T}H_{k/k-1}^{T},\\ {\hat{X}}_{k}={\hat{X}}_{k/k-1}+K_{k}\left(Z_{k}-H_{k}{\hat{X}}_{k/k-1}\right). \end{array}$$

### 3.2. BP Neural Network Algorithm

#### 3.2.1. BP Neural Network Topology

**Figure 6:**
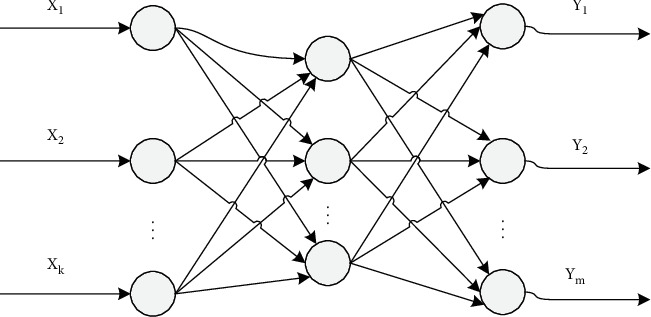
BP network model.

The BP model passes through a multilayer feedforward neural network. The BP network has multilayer hidden units. Most neural networks adopt the change form of the BP network. It is different from perceptron in the network structure. Signal forward transmission is the main feature of the BP network, which is shown in Figure 6.

The realization steps of the BP network can be obtained from the topological structure diagram of the BP three-layer neural network. First, initialize the network, provide training samples, calculate the output layer by layer from the input layer, such as the hidden layer and the output layer, carry out the vehicle output, and then, correct the weight to judge whether the prediction error reaches the error accuracy.

### 3.3. Kalman-BP Neural Network Algorithm

**Figure 7:**
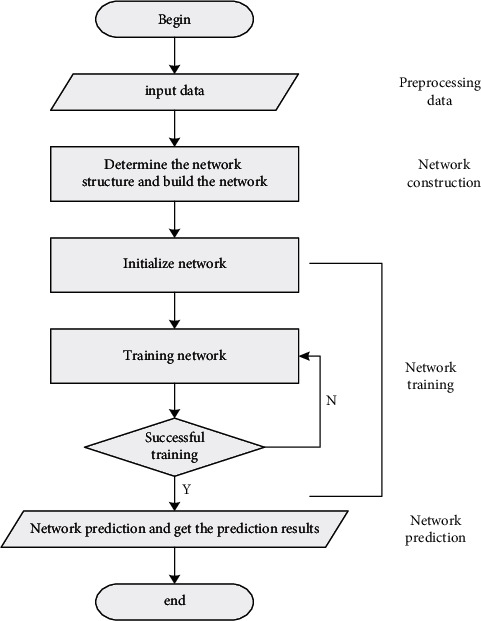
Network prediction process.

The neural network prediction method can play a great role in intelligent traffic flow prediction, and it is one of the most important methods. Similar to the neural network prediction method, our method combines the Kalman algorithm and the BP algorithm and gives full play to the advantages of the two algorithms. It mainly introduces the filtering idea into the BP network, and the performance is better after the algorithm is optimized. After the above analysis, we can get the basic flow of neural network prediction as shown in Figure 7.

The prediction process of the neural network can be divided into data preprocessing, network construction, network training, and network prediction. Data preprocessing refers to the input and normalization of data (training data and test data).

## 4. Experimental Simulation

The experimental data will collect the traffic volume observation data of a certain section of the highway in one year, one week, and one day respectively. The observation scale is one hour traffic flow, a total of 336 total data, 312 training data and 24 test data were obtained. Combining these field collected traffic flow observation data, the experiment will carry out data preprocessing, network construction, network training, and network prediction, and analyze the role of the Kalman filter algorithm in intelligent traffic flow prediction and analysis system from these four aspects.

### 4.1. Data Analysis

**Figure 8:**
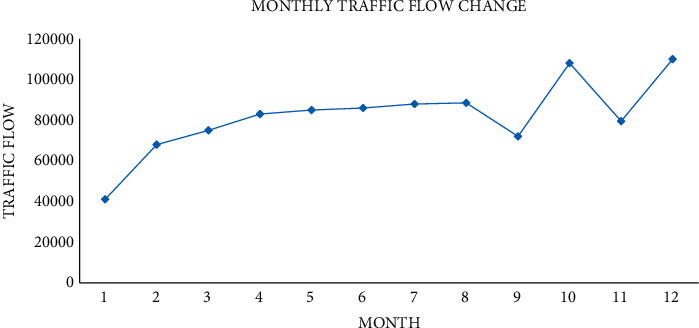
Change of traffic flow in October.

The experiment will start from one month. The changes of traffic flow from three angles of one day and one hour are analyzed. The first is the analysis of the monthly traffic flow change, as shown in Figure 8.

In Figure 8, the traffic flow increased rapidly from January to February, reaching 64000 vehicles in February. From February to August, the growth was relatively stable. It showed a downward trend from August to September and from October to November. The traffic flow from September to October and from November to December is large, and the traffic flow increases rapidly.

**Figure 9:**
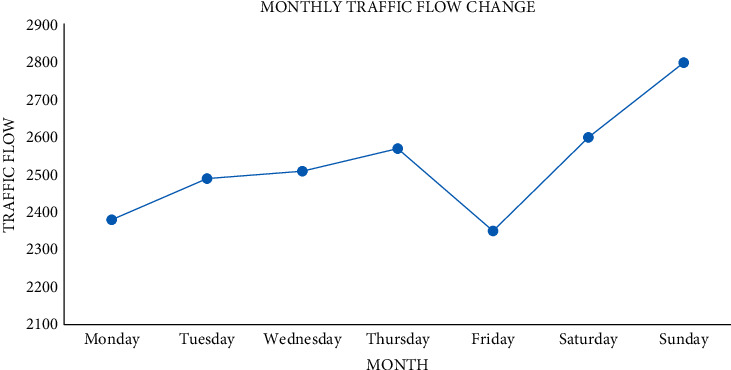
Daily traffic flow change.

Next is the analysis of daily traffic flow change, as shown in Figure 9.

**Figure 10:**
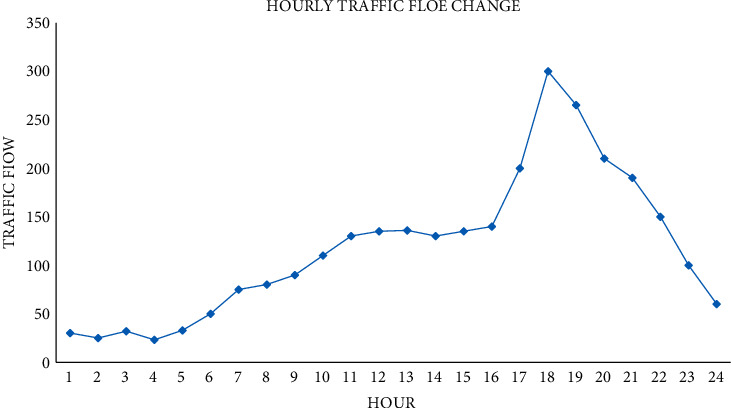
Analysis of the hourly traffic flow change.

It can be seen from the change in Figure 10 that the traffic flow increases slowly from Monday to Thursday, the traffic flow fluctuates greatly from Thursday to Sunday, the traffic flow on Friday is the least, and the traffic flow on Saturday and Sunday increases sharply.

The analysis of the hourly traffic flow change is shown in Figure 10.

In Figure 10, the hourly traffic flow in a day is less in early morning, gradually increases at 6:00, and reaches the peak from 16:00 to 19:00 with the fastest growth. It means that there is a peak period from 16:00 to 19:00 every day.

Using the traffic flow data of the above three periods as the historical reference data, traffic volume changes periodically in days. The volume first increases gradually, reaches the peak at a certain time, then decreases gradually, and oscillates before reaching the peak. In terms of peak traffic volume, it gradually increases from Monday, increases steadily and slowly on Tuesday, Wednesday, and Thursday, increases sharply on Friday and Saturday, and decreases sharply on Sunday; moreover, the total traffic volume on Friday and Saturday is also larger than usual.

### 4.2. Comparison of Prediction Error Value

**Figure 11:**
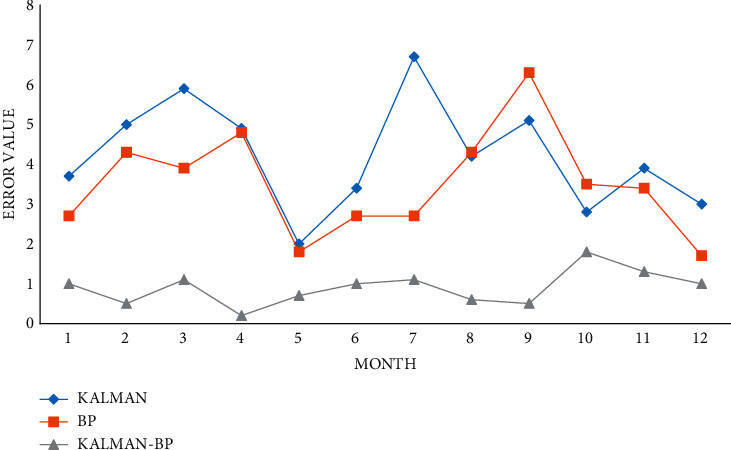
Forecast value of monthly traffic flow.

To verify the efficiency of the proposed method, this paper compares the errors between the predicted values and the measured values of the Kalman algorithm, BP algorithm, and Kalman BP algorithm, as shown in Figure 11.

**Figure 12:**
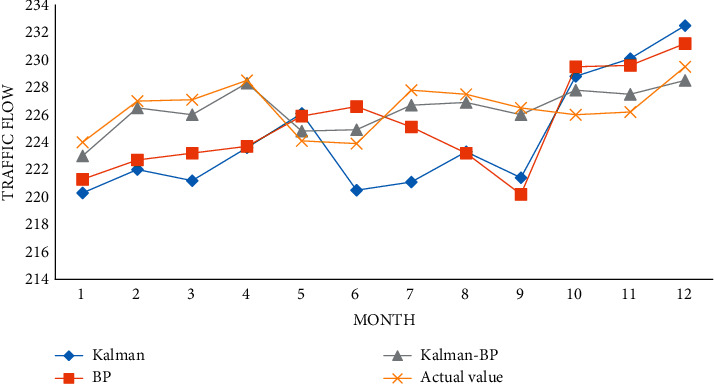
Comparison of monthly traffic flow error values.

The error value of monthly traffic flow is shown in Figure 12.

Under the monthly traffic flow prediction, the Kalman BP algorithm compared with the BP algorithm and the Kalman algorithm, the Kalman BP algorithm has the lowest error value.

### 4.3. Algorithm Operation Speed

The operation speed of the algorithm is very important. It is the basis for the algorithm. Its purity means that the system can run correctly and stably, greatly reducing the number of system maintenance, and judging the quality of a system depends on the stability of the algorithm.

**Figure 13:**
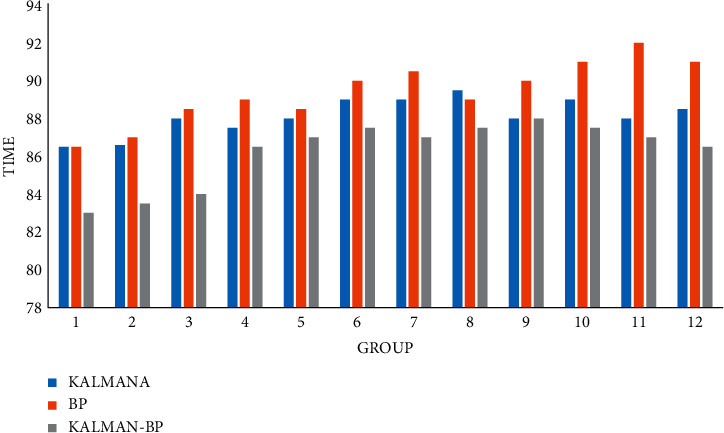
Forecast speed of monthly traffic flow.

**Figure 14:**
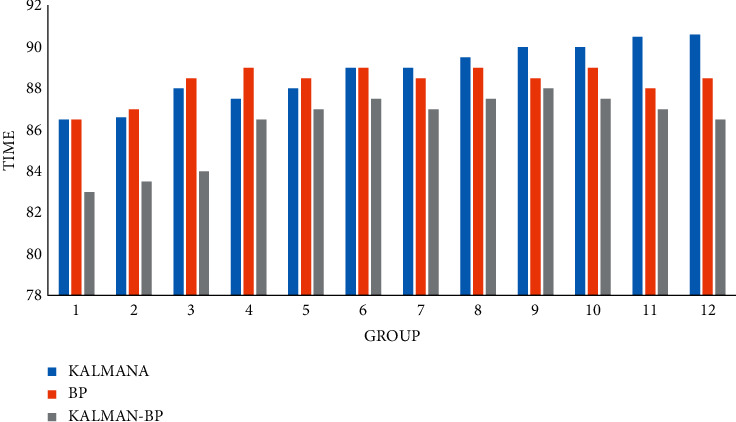
Forecast speed of daily traffic flow.

**Figure 15:**
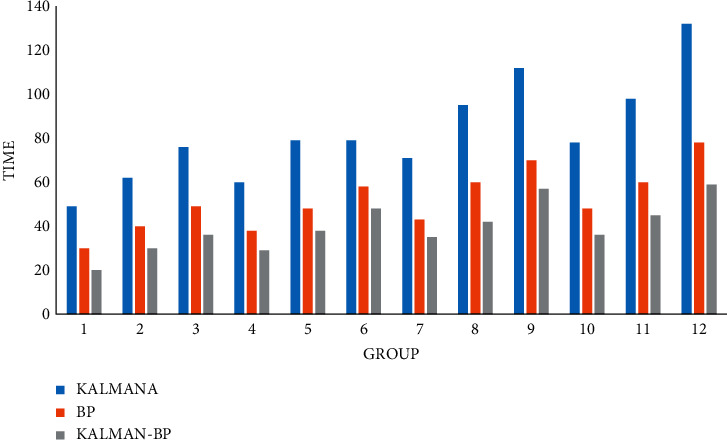
Predicted speed of hourly traffic flow.

Finally, we also compared the operation speed of traffic flow prediction of the three algorithms in three time periods, calculated in milliseconds (MS), as shown in Figures 13–15.

## 5. Conclusion

In the field of intelligent transportation, it is predicted that the current processing research stage will realize the development of intelligent transportation in smart cities. The Kalman-BP combined algorithm proposed in this paper can improve the prediction accuracy of the overall traffic flow and lower the error value. It is pointed out in this paper that there are difficulties in predictive multivariate data fusion, and higher performance optimization algorithms are needed to solve such problems. Future research works, aiming at real-time data for effective prediction, can improve the accuracy of real-time traffic prediction and lower error rate. It also considers multitask and multiplatform data real-time prediction technology supported by Internet of Things, which can be better applied to urban traffic and intelligent optimization of traffic flow.

## Data Availability

The experimental data used to support the findings of this study are available from the corresponding author upon request.
